# Arguments against Interfering with Small-Pox Inoculation, from Dr. Adams's "Inquiry into the Laws of Epidemics"
*This work being the production of one of the Editors, no impartial means of bringing it before the Public occurred but by these and the former extracts.


**Published:** 1810-01

**Authors:** 


*Arguments against interfering with Small-Pox Inoculation, from* Dr. Adams's "Inquiry into the Laws of Epidemics."*

FIRST it is shown, that before Small-pox Inoculation was known, the Faculty considered the disease inevitable once in a person's life.

"The opinion that the small-pox would necessarily occur to every one once during life, is as old as the Arabian physicians. Those who wish to learn the theories of the earliest writers, may find them collected by Diemerbroeck from Avicenna to Willis. I shall content myself with citing the last.

"It is evident that Sydenham, who seems to have fallen into the same error, had been warped by these writers in his theories of fermentation and dispumation.

"Willis's words are, —'Convenit enim homini, *omni, soli, et semel* variolis, aut morbills, affici: si forte quispiam in tota vita immunis degerit, aut alius in hos affectus sæpius inciderit, sunt hæc rara et inusitata naturæ eventa quæ communi observationi minime derogant: quin omnino rarum sit, quod nimirum *cuncti et soli* homines sint variolis

---

† This work being the production of one of the Editors, no impartial means of bringing it before the Public occurred but by these and the former extracts.

variolis et morbillis obnoxii, atque *unica plaga* iis absolvi soleant."

He afterwards assigns as the cause of this universal prædisposition to the disease, certain impurities of the blood, *inter prima fœtus rudimenta in utero concepta*; quæ [particulæ] diu delitescunt; postea a *causa evidenti* commotæ, cum sanguine fermentescunt; ipsique *ebullitionem* ac deinde *coagulationem* inducunt; è quibus plurima hujus morbi symptomata oriuntur.'

"*Causa evidens*, quæ hæc semina fermentativa commovet, et sæpissime in actum deducit, triplex assignatur: scilicet *contagium* aliunde susceptum, *dispositio aëris*, ac immodica *sanguinis et humorum perturbatio*.'

"Gentilis and Mercurialis differed from this opinion of the cause of impurities, but still consider small-pox as morbum hæreditarium atque hinc nullum fere hominem ab iis immunem esse posse quid omnes nascuntur ex parentibus hoc vitio contaminatis.

"Fernelius derives the disease à causa quadam cœlesti et occulta cui cum infantes et pueri minus resistant quam adulti, hinc fieri quod illi frequentiores variolis laborent quam hi.

"Sennertus differs from these last writers, without assigning any better cause than the humidity of spring and autumn with vitiated particles of the blood.

"Diemerbroeck shows the folly of this notion of impurities, by inquiring how any person could escape from such an effect, when all must be subjected to the cause. He instances a great number of his relations who had lived to a very advanced age, without suffering the disease; and, lastly, himself, who, on the verge of seventy, after attending thousands without any caution, had escaped.— But this would rather make in favour of Willis's theory, who expressly says, that if perchance an individual should escape during life, or another should fall into the disease more than once, such uncommon events do not invalidate a general law of Nature. Now it is plain, that Diemerbroeck did not ascribe his security to a want of exposure, it could therefore only be accounted for by that peculiarity of constitution which Willis has remarked, as not inconsistent with his theory. This want of susceptibility of small-pox is, I am persuaded, much more rare than is generally suspected. Among the infants inoculated at the hospital, the number is too small to ascribe to any other cause than some temporary affection, which occupies the constitution at the time. Among adults, the number number is greater; and here we generally find that the subject has remained with his brothers and sisters or play-mates, whilst under small-pox. Under these circumstances, it is probable, an accidental inoculation has taken place, and the disease has passed off so mildly, that the attention to the greater sufferers has absorbed the whole anxiety of the family. But I seem to have forgotten that my only intention was to show, how much the opinion of the necessary universality of the disease was encouraged by the profession. Diemerbroeck, we may see, though he discourages the opinion, does not consider exposure to the contagion, as necessary to induce the disease. It however does him credit, that he professes himself unable to assign any cause whatever. 'Quis enim (he concludes) tantæ rei se veram et perceptibilem rationem daturum promittet? Hæc quippe sunt ex illis arcanis quorum causas nos exacte scirenoluit altissimus Conditor.'—Diemerbroeck de Variolis, p. 275. I do not recollect any writer, before the introduction of inoculation, who proposes any means of escape which human prudence can advise in a populous town."

Secondly, the Author shows, that the disease has not, as is generally urged, increased in fatality since the introduction of that practice.

"The only plausible argument that remains against permitting small-pox inoculation in the metropolis, is, that the number of deaths by that disease has increased since the practice was introduced. Even if this were true, we have shown that the increase has been much more considerable in the other contagions; and Dr. Heberden remarks, that the number of sudden deaths have doubled during the last century. It will easily be seen, that if more children are reared, which the same authority admits; and if fewer die of the diseases of poverty, more must be exposed to those causes of fatality from which no improvement in life can secure us, or which, like apoplexy, are more common in the advanced stages of life. From this we might expect that the number of victims to small-pox would have been in a ratio equal to its greater fatality above the measles; and such would probably have been the case had not inoculation been introduced. Those who estimate the probable number of deaths from small-pox by the probable number who may be exposed to the disease, must be erroneous as far as relates to the metropolis, where all are exposed; and, if we may believe Sydenham, all are infected during an epidemic. The only calculation calculation we can make, is by estimating the number residing in the metropolis, that are susceptible of the disease. In this manner we find Dr. Willan accounting for the progress of the disease in the years 1796, 1797, and 1798. During the first of these years happened the severest epidemic small-pox recorded since 1757. During the second, the number was reduced to 522, which is fewer than at any period since, and for several years before; during the third, the numbers were again high. On this occasion that accurate writer remarks, 'The new subjects for this increased mortality must have been produced by the births within the two years, and by the influx of adult persons from the country, who never had the small-pox, to the amount of several hundreds every year.'

"This last circumstance would be sufficient to account for the increase of deaths by small-pox since inoculation, had such really taken place; because, since the rich have lost their terrors by inoculation, they have been too inattentive in receiving domestics who have not passed thro' the disease. There appears also an inaccuracy. The estimate has usually been made by *the proportion of deaths from all diseases*, without sufficiently reflecting that the numbers who formerly died in early infancy, swelled the total of burials, without adding to the real population.— Dr. Heberden, willing to view the question in every light, has given what he calls a coarse statement from an average of about ten years, at the beginning, middle, and end of the 18th century, selecting such years in which the whole number of deaths was nearly the same, viz. about 21,000. By this mode of calculating, he finds the increased mortality by small-pox to be from 1600 in the beginning to 2000 in the middle and end of the century.— This mode is objectionable, because certain years of particular epidemics may produce extraordinary mortality, and may supersede small-pox as an epidemic. Hence, in the succeeding years, a greater number will remain susceptible of small-pox, yet the general mortality may be less. Thus the decade from 41 to 50 inclusive, was the most fatal in fever, yet the number of deaths by small-pox was particularly low. It appears, therefore, that to make a fair statement where we cannot suppose the population lessened, we ought to take the whole numbers as they are, without regarding the greater or less mortality of particular years, excepting what are called the plague years, none of which happily have occurred during the last century. In distributing the century into three parts,

I shall

I shall for convenience, select the last 90 years, being more easily divided into three thirties. The first decad is omitted, not only on account of the order which it enables us to maintain, but because the mortality by fevers as well as small-pox and measles was so low, for that early period, as can only be accounted for by a less population.

"According to the proposed division, then, we shall find that there died of small-pox in the thirty years,

| | | |
|---|---|---|
| From 1711 to 1740 inclusive | - | 65383 |
| From 1741 to 1770 ditto | - - | 63308 |
| From 1771 to 1800 ditto | - - | 57268; |

making the number of deaths by small-pox greater in the first thirty years by 2075, than in the second thirty years, during which inoculation had acquired some stability, and greater by 8115 than in the last thirty years, during which inoculation was the established practice of most prudent families.

"At the same time, the mortality by measles has taken a quite different course, the number of deaths in the second period of the same ninety years being greater than in the first by 1781, and the number in the last thirty years exceeding the first by 2018.

"By taking the estimate of these two diseases, we save the necessity of any estimate concerning an increased population, which is very uncertain. For how much soever the population of the town may have increased, we are not able to ascertain how much of this increase is confined to Mary-le-bone and Pancras, which are not included in the bills, nor whether the depopulation by the improvement of London, withiu the walls, may not equal the increase of the other parishes included within the bills.

"Imperfect as these bills are, whilst we have no other records, it is justifiable to refer to them, and, as Dr. Heberden remarks, taken on a large scale, they are not likely to deceive us much. But it is truly painful to see men, whose intentions are honest, led astray by the artful quotation of single years. In 1804, we are told that vaccination had so far reduced the number of deaths by small-pox, that they amounted to only 622. It might easily be answered, that in 1797, they amounted to only 522. What was the cause of each? That there were fewer inhabitants in London susceptible of the disease in 97, in consequence of a previous epidemic; and in 1804, in consequence consequence of the zeal for vaccination. All this is highly in favour of vaccination, but makes nothing towards the extermination of small-pox, which can only be kept under by inoculation or vaccination.

"In the year 1805, it is added by these honest zealots, the number of deaths by small-pox increased from 622 to 1685, in consequence of the difficulties thrown in the way of vaccination. But it is notorious that those difficulties increased during the years 1806, 7, and 8, yet the average number of deaths in those three years has been only 1280, which is less than in any three successive years that can be produced within the last ninety years of the preceding century, or than any single year, not preceded or followed by an epidemic small-pox. During the two first and part of the last of these years, small-pox inoculation increased to such a degree, as to alarm many well intentioned people. Yet part of the previous exemption might be imputed to inoculation, as the deaths by small-pox have increased since that practice was discontined at the hospital. At the same time, I conceive vaccination has had a very great share in decreasing the deaths by small-pox; not so much by what has been done in London, but because during the early zeal in the country, so many were vaccinated who, by this time, have resorted to the metropolis, and if they had not been prevailed on to vaccinate, would probably have arrived susceptible of small-pox.

These passages are selected as the most detached, being thrown into Notes at the end and into the Appendix. They serve as additional proofs of what is advanced in the text; namely, that before inoculation was known, the disease was considered inevitable in the larger towns; and that since the introduction of that practice, the number of victims has lessened in London.

*Memoir*

